# Supplementary figures and images for: CoSTA: unsupervised convolutional neural network learning for spatial transcriptomics analysis
Source: BMC Bioinformatics. 2021 Aug 9;22:397. doi: 10.1186/s12859-021-04314-1 (PMC8351440; doi:10.1186/s12859-021-04314-1)

Fig. S1

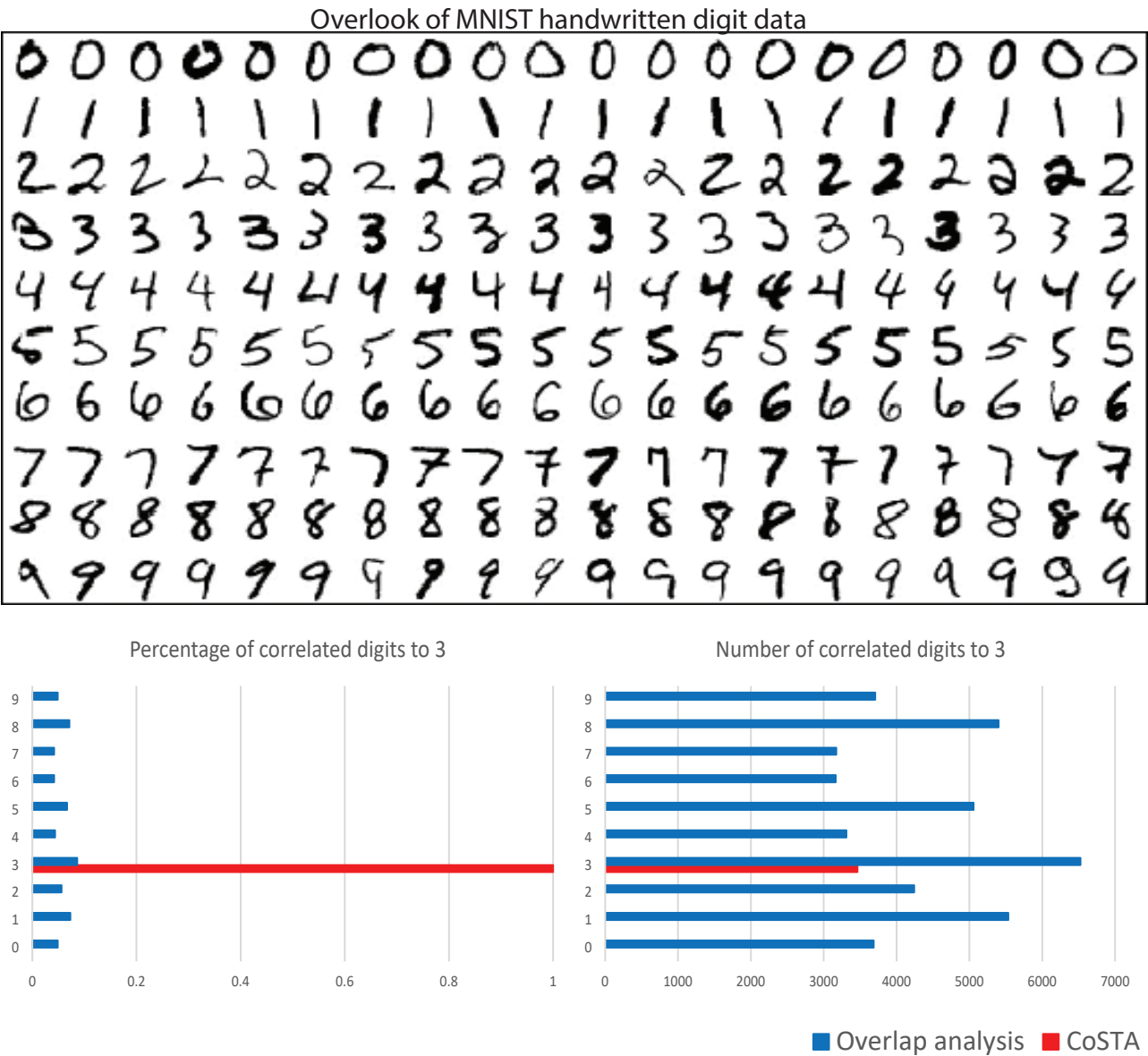

Supplement: Supplementary file 1 — Additional file 1. Supplementary Fig. 1: Comparison of CoSTA and overlap analysis performance in finding correlated digits to digit 3. 1000 images are sampled from the full MNIST dataset, and each digit contains 100 samples. CoSTA (red bars) uniquely calls samples of digit 3 as correlated to digit 3. However, overlap analysis (blue bars) identifies some instances of all digits as showing some overlap with digit 3. CoSTA is more specific, but less sensitive: CoSTA reports a smaller number of correlated digit 3 images (bottom right) while overlap analysis reports a greater number of correlated digits overall. [file 12859_2021_4314_MOESM1_ESM.pdf]

Fig. S2

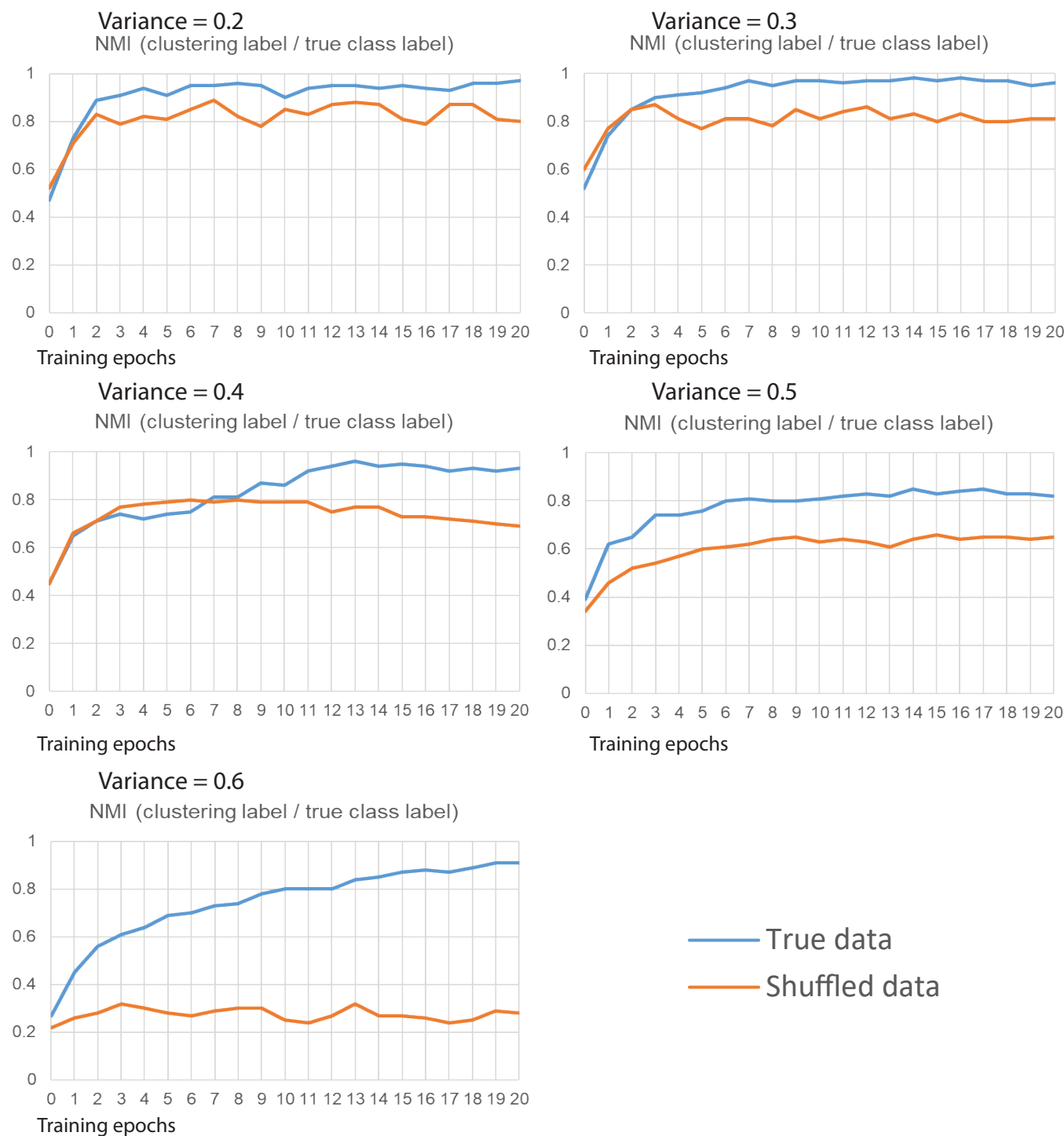

Shuffled patterns

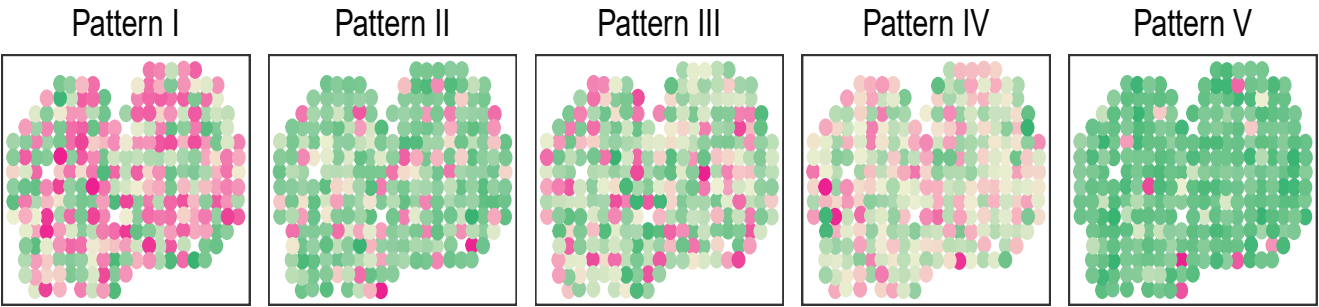

Supplement: Supplementary file 2 — Additional file 2. Supplementary Fig. 2: Learning curves of CoSTA using true and shuffled synthetic datasets. 2,000 simulated gene matrices were used for each pattern, as in Fig. 1, with different levels of noise added (“variance”). Shuffling for each pattern and each simulated gene was performed identically so that pixelwise correlations were preserved but spatial relationships between neighbors were disrupted. (see Methods and Fig. S12 for shuffling approach details) NMI compares the clustering labels generated by CoSTA against the true class label. [file 12859_2021_4314_MOESM2_ESM.pdf]

Fig. S3

A

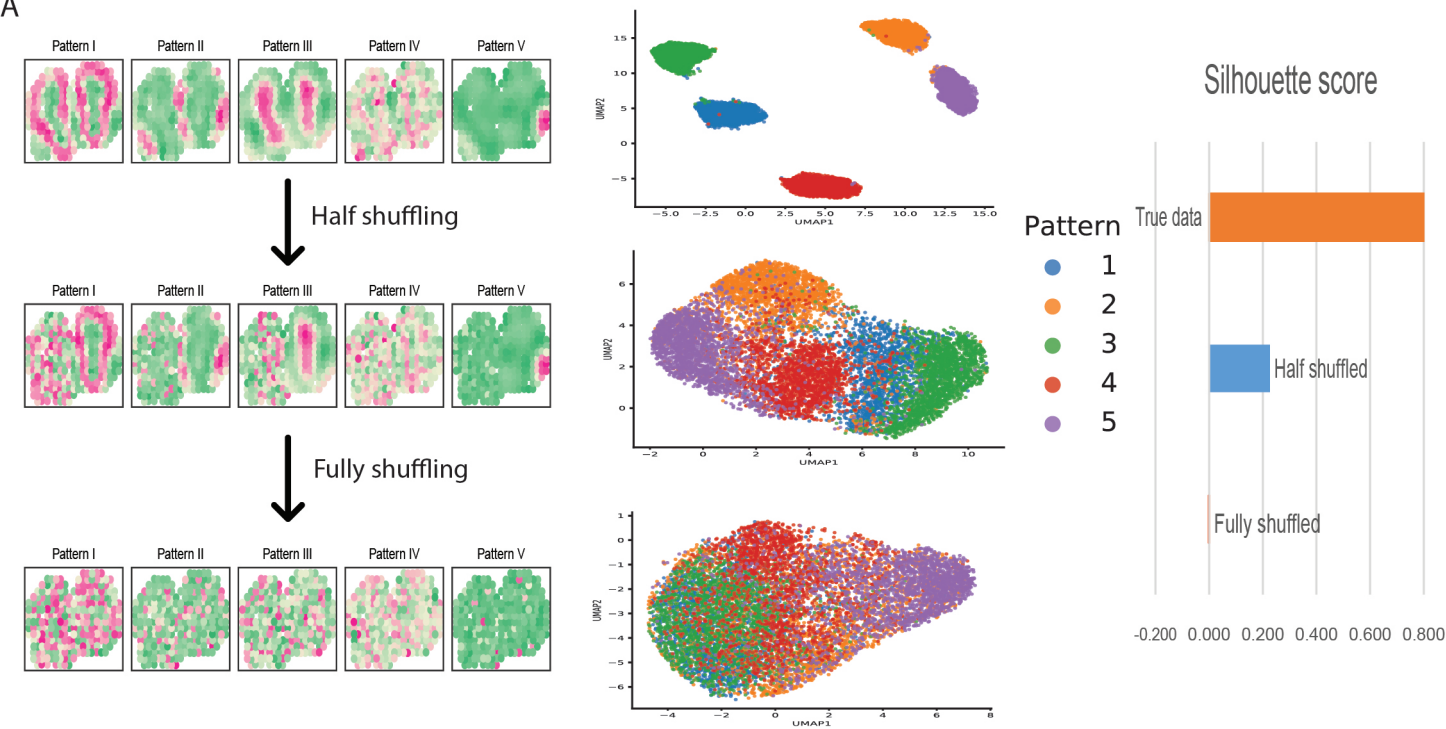

B

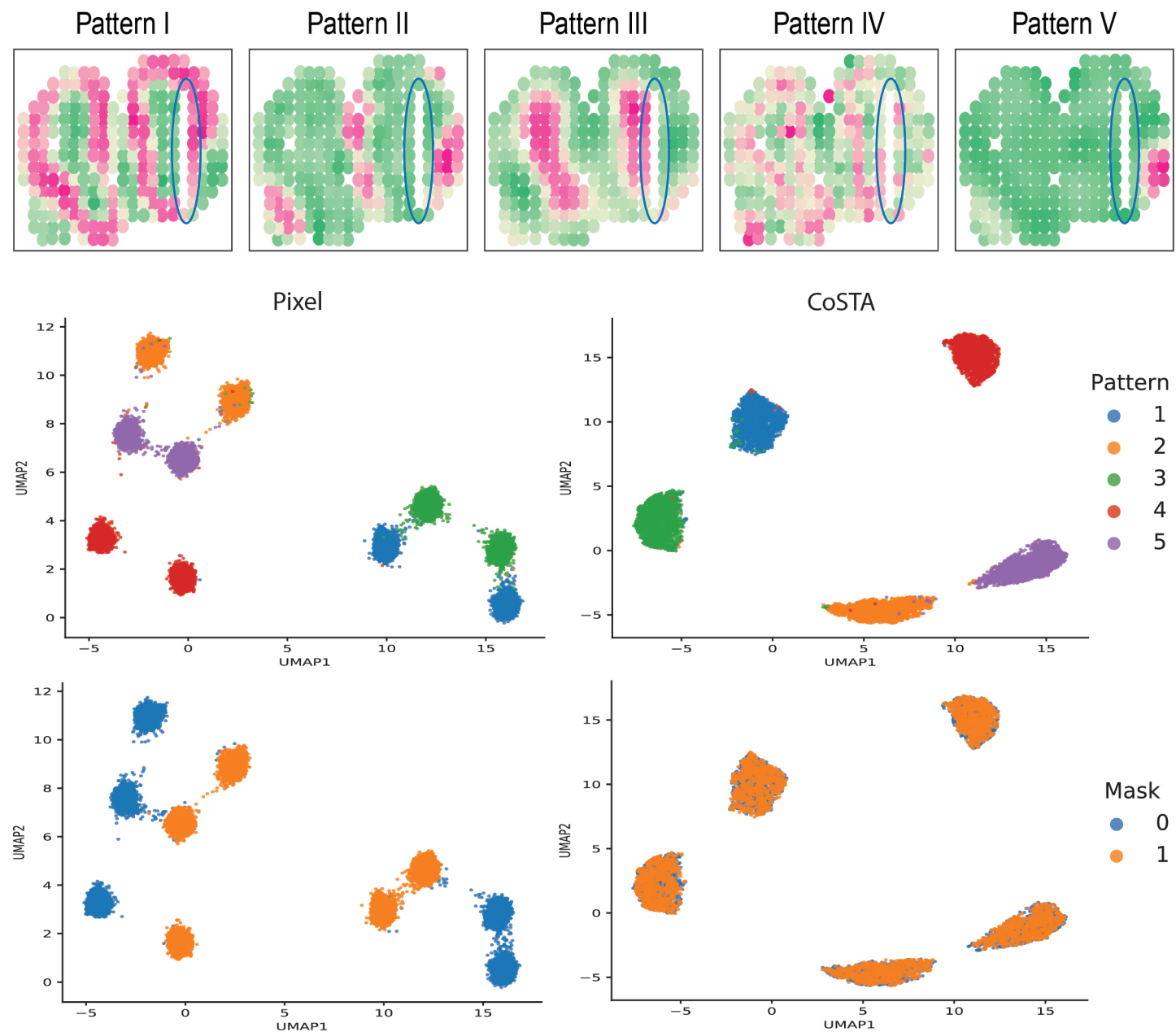

Supplement: Supplementary file 3 — Additional file 3. Supplementary Fig. 3: Performance of CoSTA using synthetic datasets with perturbations. A) Left: the same initial spatial patterns as in Fig. 1 were used. CoSTA was applied to classify 2,000 simulated genes for each pattern from the original patterns (top), half shuffled (middle), and fully shuffled (bottom) patterns. Applying trained CoSTA representations of simulated genes are visualized by using spatial representation in 2D UMAP. Genes are colored based on the true synthetic pattern from which they are derived. Silhouette scores quantify how well the representation distinguishes different patterns. (Closer to 1 = more distinguishable patterns are recovered). B) Disruption test through masking. Half of the simulated genes from each pattern have a masked region, simulating experimental missing data. The masked region is circled in blue in the upper panel. Representation of simulated genes based on pixelwise values (left) and features extracted by CoSTA (right) are visualized in 2D UMAP, and genes are colored based on pattern type from which they were generated (upper panel) or according to whether they belonged to the masked or unmasked set (lower panel). [file 12859_2021_4314_MOESM3_ESM.pdf]

Fig. S4

## Examples of non-SE genes

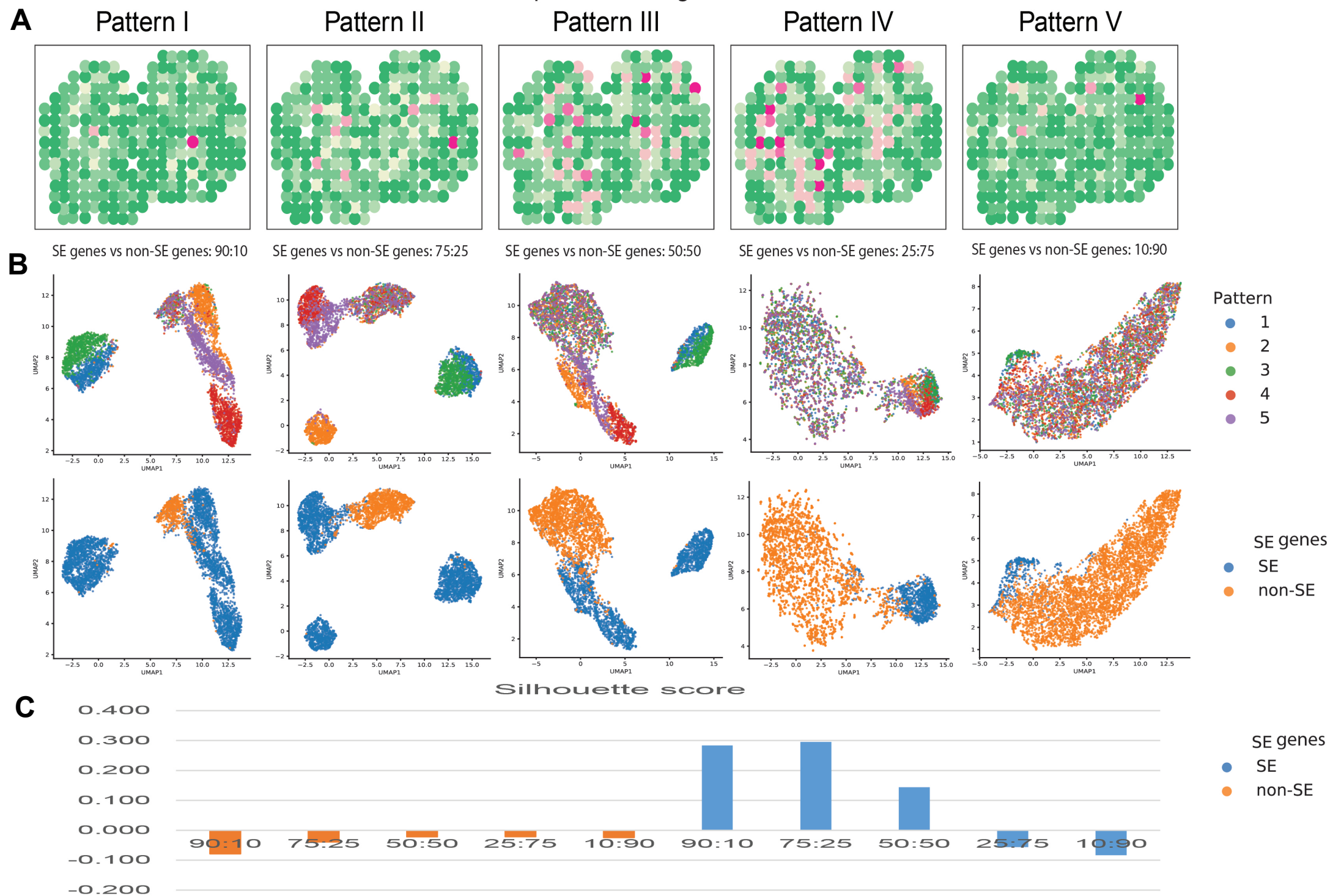

Supplement: Supplementary file 4 — Additional file 4. Supplementary Fig. 4: Training CoSTA with different ratios of SE and non-SE genes. (A) To simulate non-SE genes, five patterns without clear spatial features were used. (B) Simulated non-SE genes were mixed with SE genes simulated from the 5 patterns in Figure 1 in different ratios from 90:10 to 10:90. CoSTA representations of these gene mixtures are visualized in 2D UMAP. Genes are colored based on pattern membership (top) or SE type (bottom). (C) Silhouette scores quantify how well the representation distinguishes different patterns for SE and non-SE genes across different mixture ratios. [file 12859_2021_4314_MOESM4_ESM.pdf]

Fig. S5

10 nearest neighbors

20 nearest neighbors

50 nearest neighbors

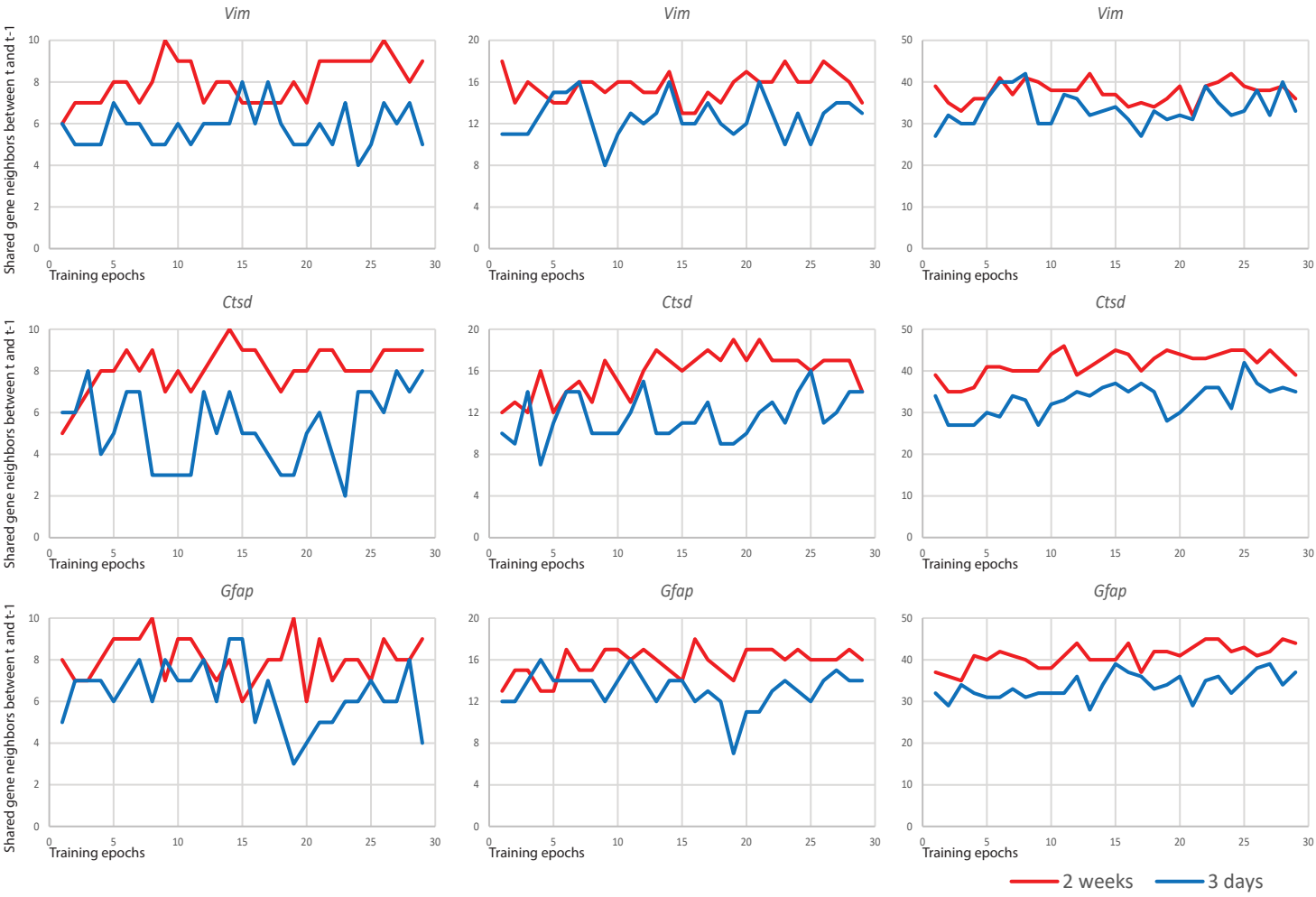

Supplement: Supplementary file 5 — Additional file 5. Supplementary Fig. 5: The number of overlapped neighbors of Vim, Ctsd, and Gfap before and after each weight updating across all epochs, considering either 10 nearest neighbors (left), 20 nearest neighbors (center), or 50 nearest neighbors (right). [file 12859_2021_4314_MOESM5_ESM.pdf]

Fig. S6

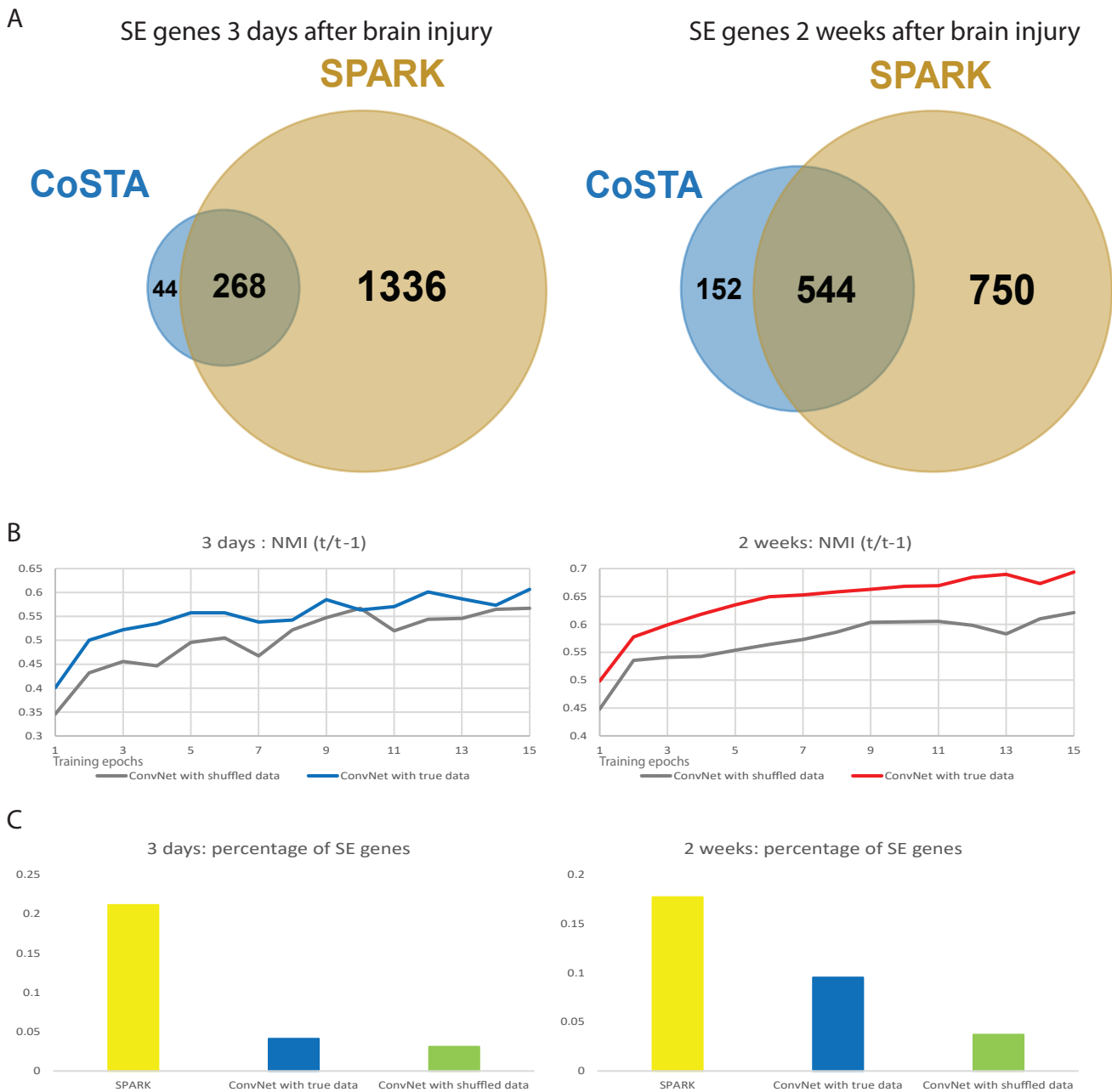

Supplement: Supplementary file 6 — Additional file 6. Supplementary Fig. 6: The number of SE genes after 3 days and 2 weeks brain injury. (A) Overlap of SE genes identified by SPARK or CoSTA. (B) Learning curve of CoSTA with original and shuffled data. (see Methods and Fig. S12 for shuffling approach details) Y-axis shows NMI calculated between cluster labels at training epoch t and cluster labels at previous epoch t-1. X-axis shows training epoch t. (C) Percent of all measured genes that are called SE genes by the 3 approaches. [file 12859_2021_4314_MOESM6_ESM.pdf]

Fig. S7

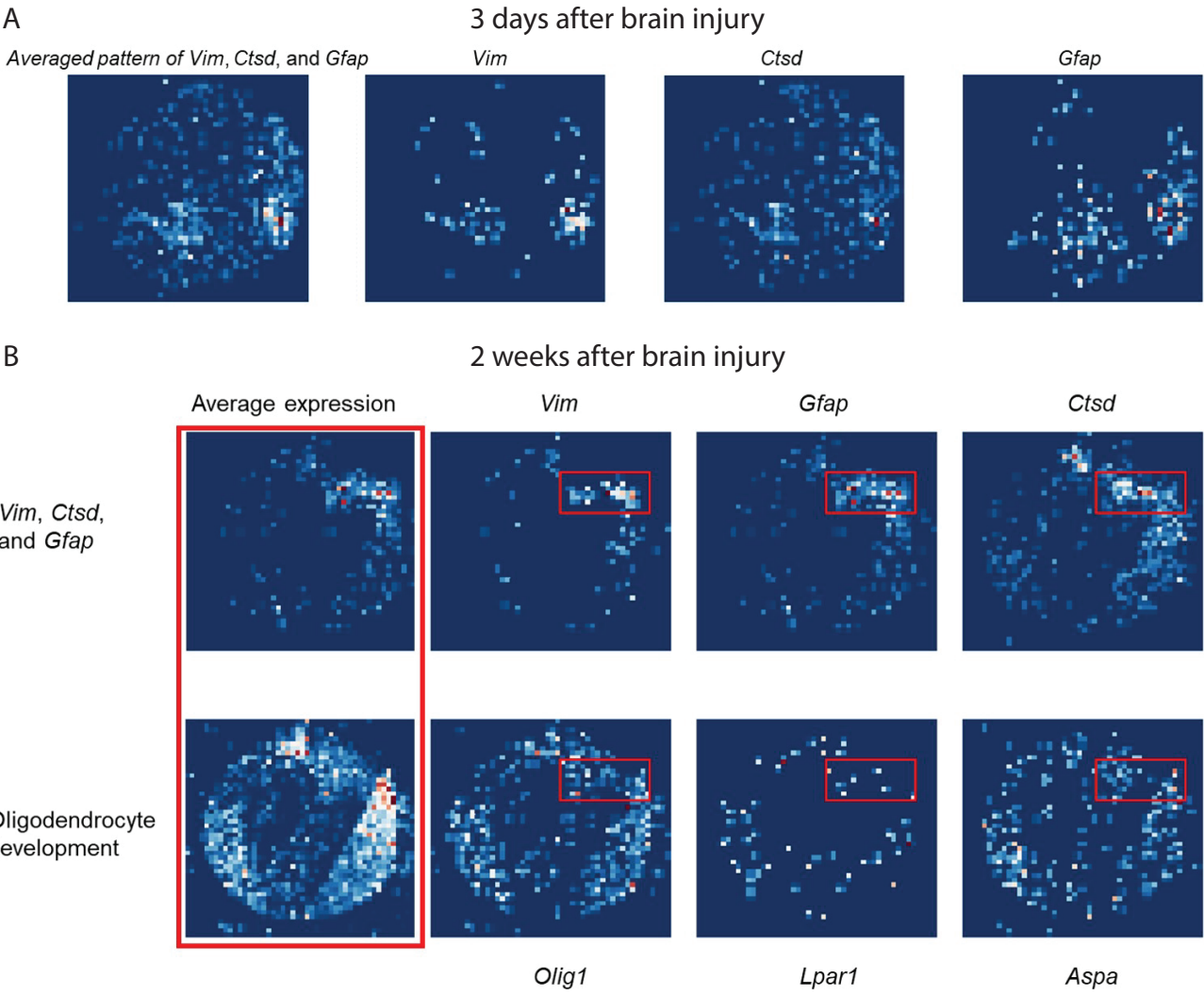

Supplement: Supplementary file 7 — Additional file 7. Supplementary Fig. 7: Expression patterns of Vim, Ctsd, and Gfap 3 days and 2 weeks after brain injury. (A) Expression patterns of Vim, Ctsd, and Gfap 3 days after brain injury. (B) Expression patterns of Vim, Ctsd, Gfap and genes involved in oligodendrocyte development (bottom row) 2 weeks after brain injury. Patterns that are visibly similar between Vim, Gfap, and Ctsd (small red boxes) are not strikingly visible in oligodendrocyte development genes. [file 12859_2021_4314_MOESM7_ESM.pdf]

Fig. S9

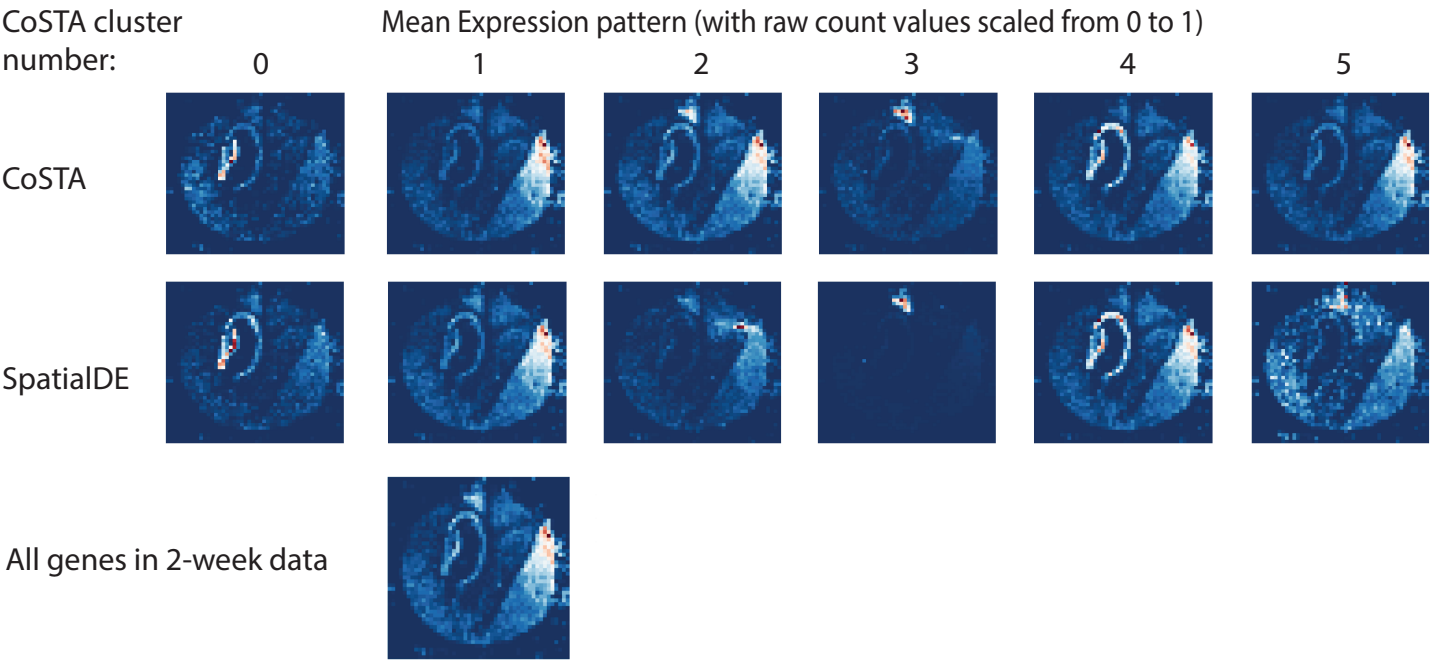

Supplement: Supplementary file 9 — Additional file 9. Supplementary Fig. 9: Expression patterns of SE genes identified by CoSTA 2 weeks after brain injury. SE genes were clustered into 6 groups by SpatialDE and CoSTA. CoSTA cluster numbers correspond to Figure 4d and the most similar SpatialDE cluster is placed below the most closely corresponding CoSTA cluster when possible. The SpatialDE cluster containing Vim, Gfap, and Ctsd is cluster 2. Average expression pattern in 3rd row shows the overall pattern of all genes combined in the 2-week dataset. [file 12859_2021_4314_MOESM9_ESM.pdf]

Fig. S10

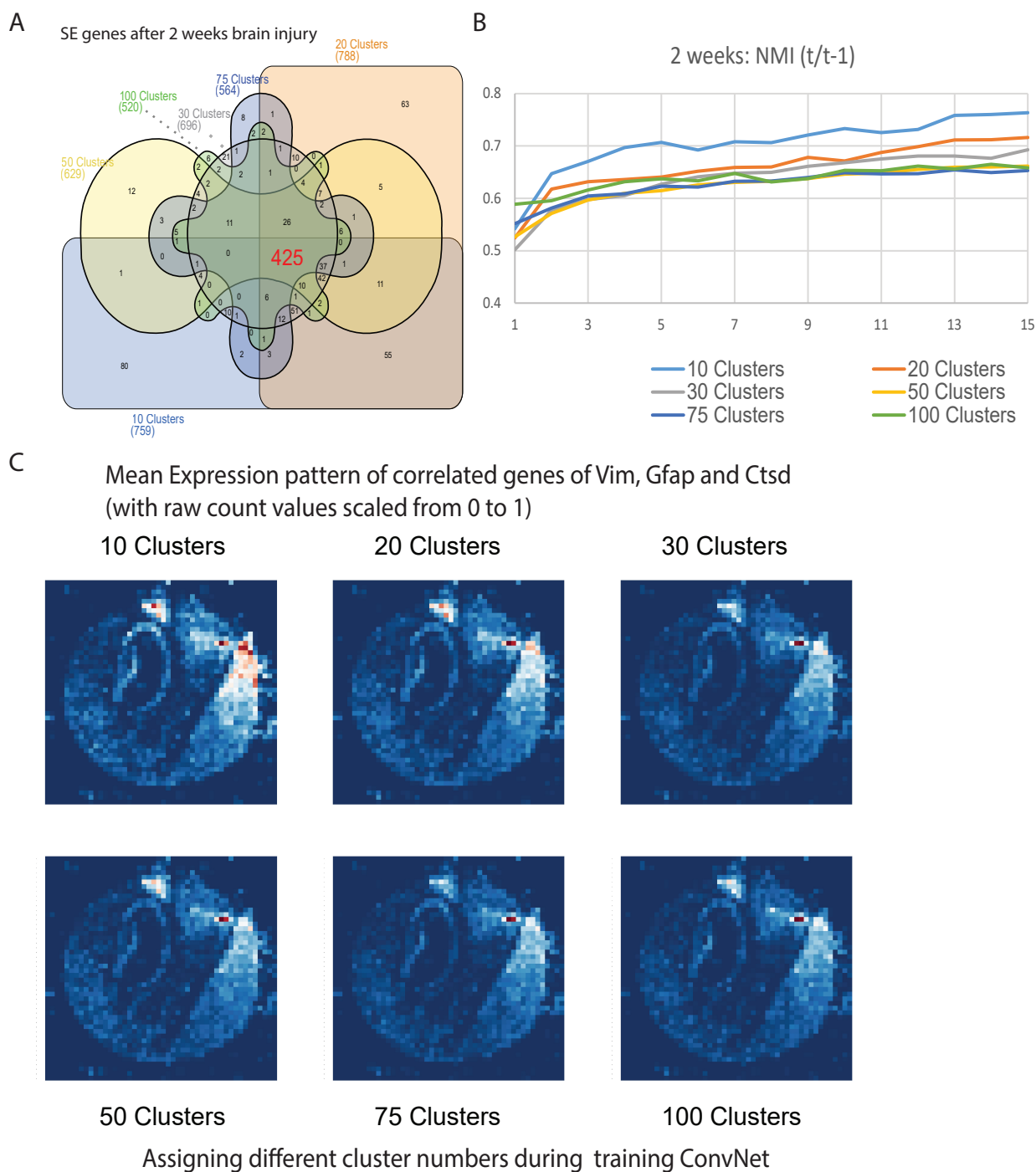

Supplement: Supplementary file 10 — Additional file 10. Supplementary Fig. 10: Effect of cluster number on CoSTA results with 2-week post injury Slide-seq data. a, SE genes identified by CoSTA with 10-100 clusters. b, CoSTA learning curve with 10-100 clusters. Y-axis shows NMI calculated between cluster labels at training epoch t and cluster labels at previous epoch t-1. X-axis shows training epoch t. c, Mean expression pattern of genes found to be correlated with Vim, Gfap and Ctsd identified by CoSTA with cluster numbers ranging from 10-100. Raw count values are scaled from 0 to 1 for these visualizations. [file 12859_2021_4314_MOESM10_ESM.pdf]

Fig. S11

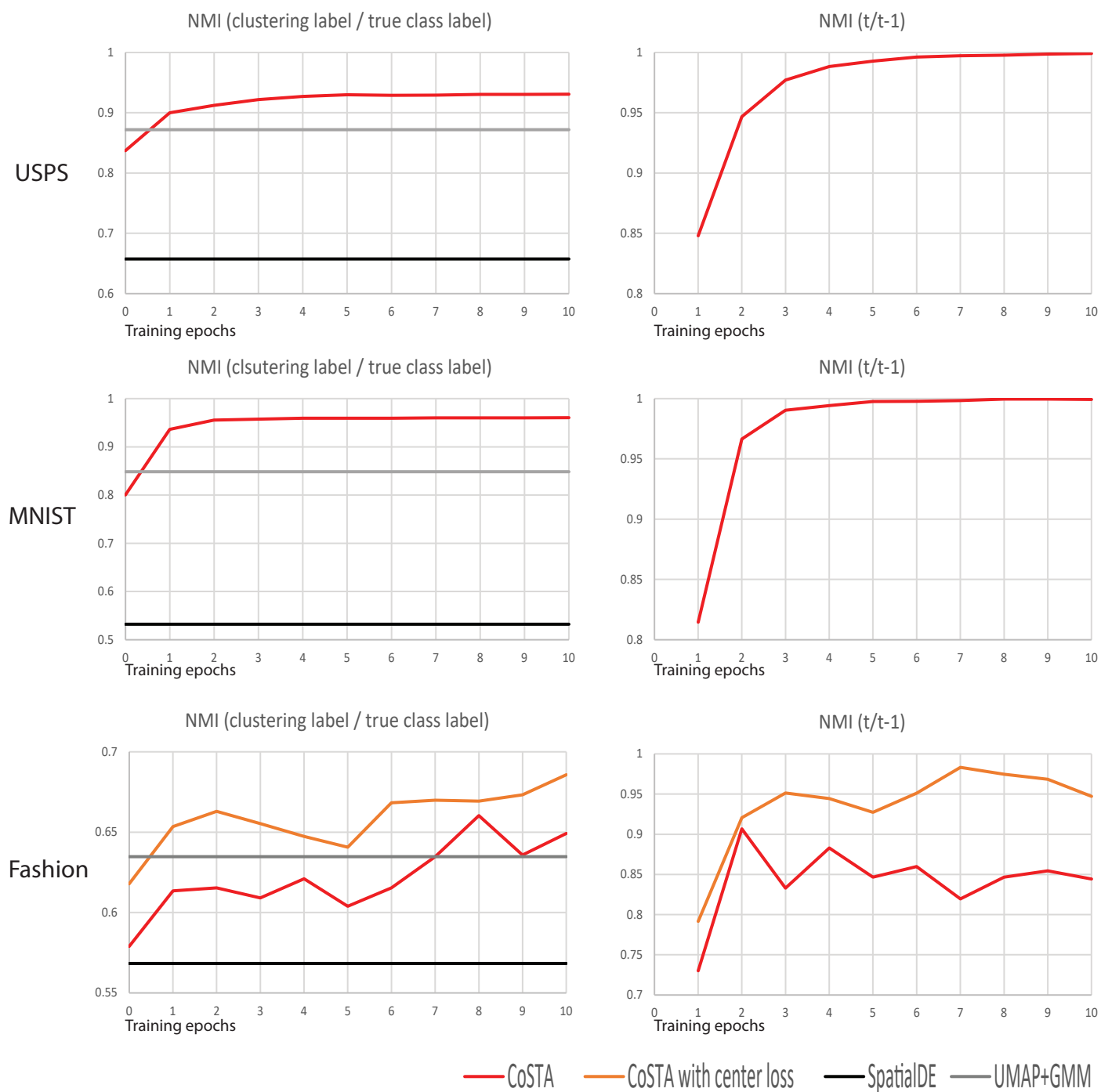

Supplement: Supplementary file 11 — Additional file 11. Supplementary Fig. 11: CoSTA approach applied to clustering USPS, MNIST and Fashion datasets. Left panels: Models were trained for 10 epochs. After each weight updating, we clustered images into 10 clusters and directly compared them to true class labels through NMI. The grey line indicates clustering by UMAP+GMM with pixel values as features. The black line indicates clustering by SpatialDE. The orange line represents learning with combined center loss and bi-tempered logistic loss in Fashion dataset. Right panels: NMIs between clustering at the tth updating and the previous (t-1)th updating. [file 12859_2021_4314_MOESM11_ESM.pdf]

**A**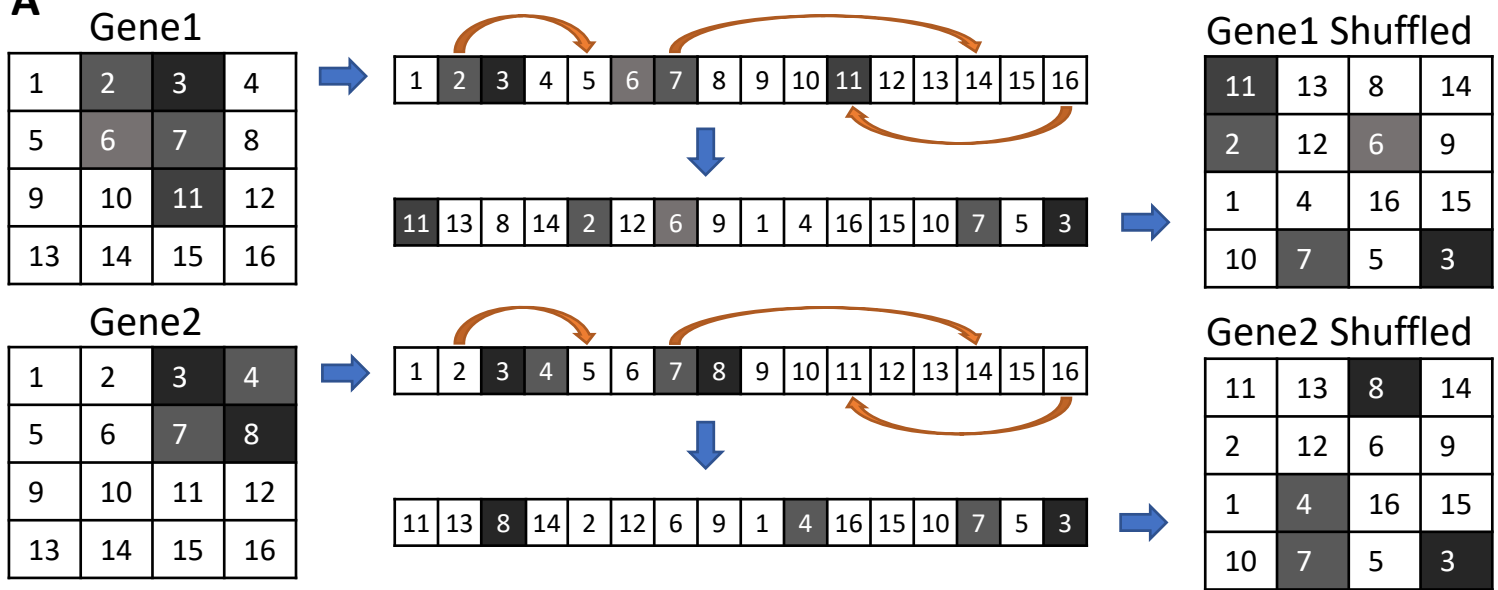**B**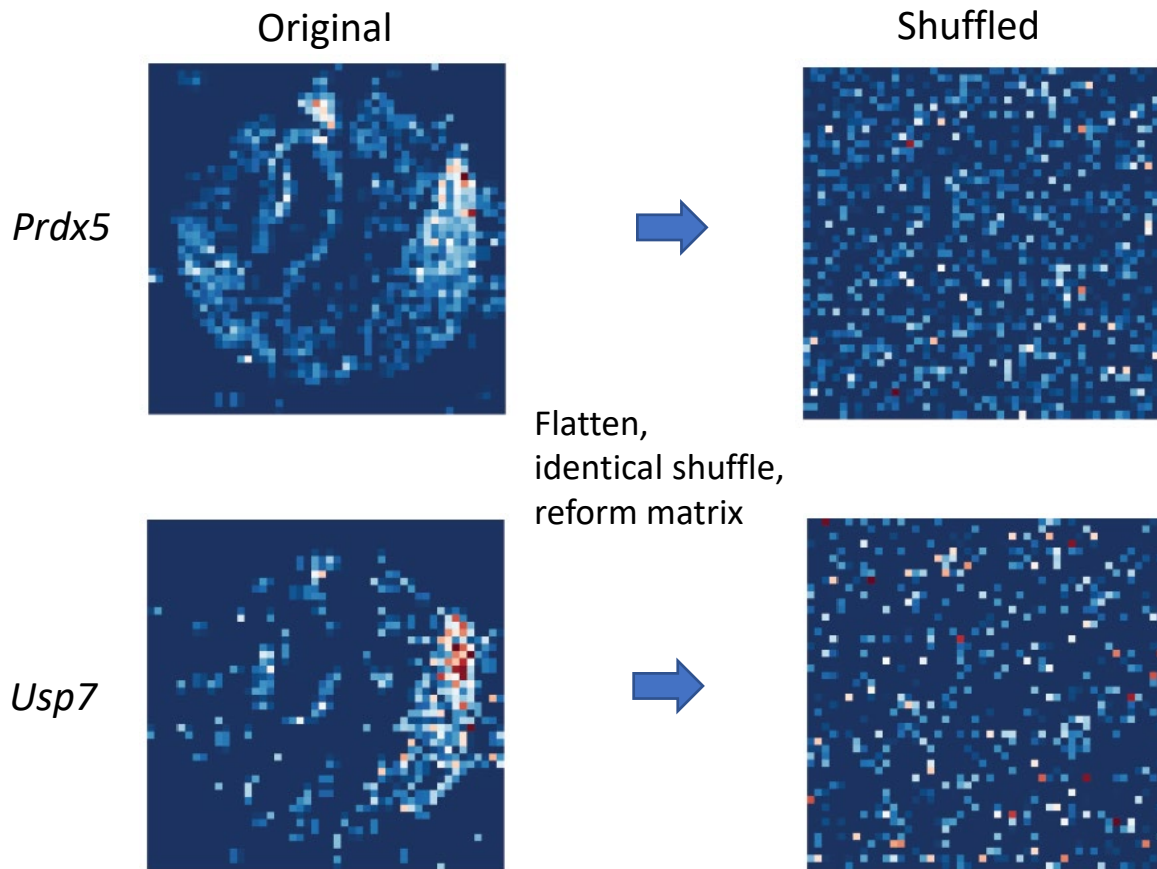

Supplement: Supplementary file 12 — Additional file 12. Supplementary Fig. 12: Shuffling approach to preserve pixel correlation but disrupt spatial information. (A) Cartoon representing shuffling approach. Right: two initial 4x4 gene matrices (dimensions are small for example purposes). Each matrix shows a certain pattern of expression where a cluster of neighboring pixels show similar gene expression. Pixels are numbered so their positions can be tracked through the shuffling process. Middle: the 4x4 matrix is flattened into a single vector and then the positions of pixels are shuffled in the same way for Gene1 and Gene2 (orange arrows show a few example pixel rearrangements). The pixel ordering within each image is disrupted but each gene shares the same pixel ordering with other genes. This preserves individual pixel correlations across images from different genes but disrupts the spatial ordering and relationships between neighboring pixels. Right: shuffled vectors are reformed into a 4x4 matrix. (B) Example of shuffling result for 2 example Slide-seq gene matrices. Left: original gene expression image matrices. Shuffling is applied identically to the two genes as shown in A. Right: Resulting shuffled matrices. Visible spatial patterns are gone, but the pixel correlation of the two images would remain the same. [file 12859_2021_4314_MOESM12_ESM.pdf]
