# Supplementary material for: CoSTA: unsupervised convolutional neural network learning for spatial transcriptomics analysis
Source: BMC Bioinformatics. 2021 Aug 9;22:397. doi: 10.1186/s12859-021-04314-1 (PMC8351440; doi:10.1186/s12859-021-04314-1)

Fig. S8

A

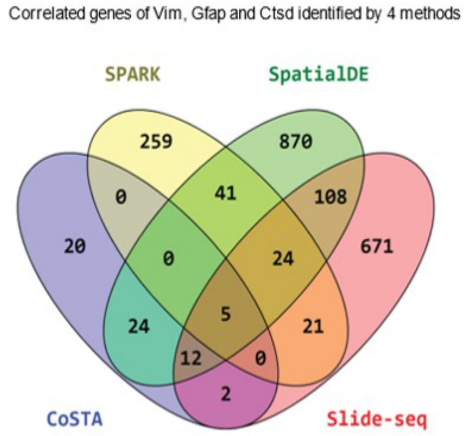

B

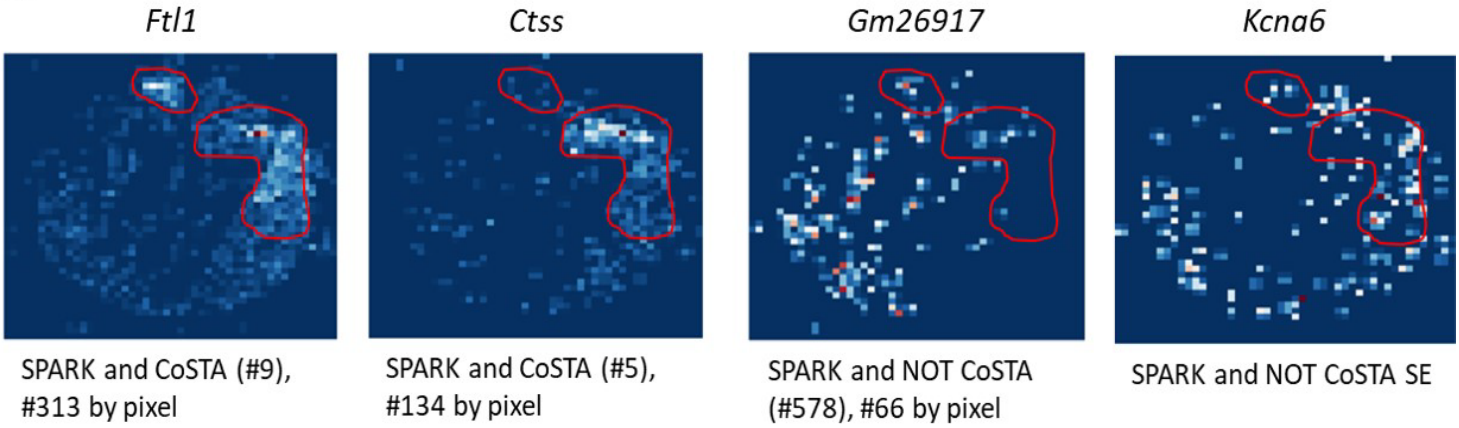

C

GO biological process	Fold Enrichment	FDR
collagen metabolic process	89.70	0.008
RNA metabolic process	1.41	0.079
Intermediate filament organization	149.5	0.18
Regulation of vascular endothelial growth factor signaling pathway	74.75	0.26
Astrocyte differentiation	49.83	0.24

D

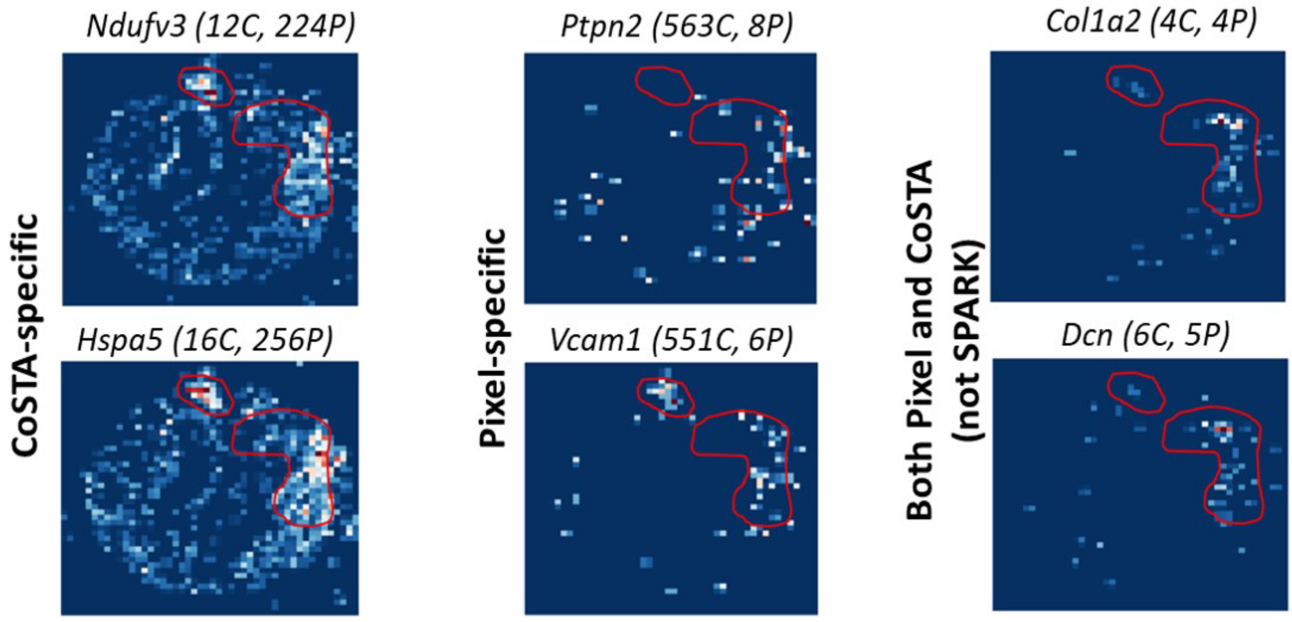

Supplement: Supplementary file 8 — Additional file 8. Supplementary Fig. 8: Comparison of SPARK, SpatialDE, CoSTA, and pixel overlap results. (A) Overlap of gene lists correlated with Vim, Ctsd, and Gfap at 2 weeks after injury identified by CoSTA, SPARK, SpatialDE, and overlap analysis (“Slide-seq”). (B) Examples of gene expression images for genes detected as similar to Vim, Gfap, and Ctsd by SPARK and also by CoSTA (left) or SPARK and not CoSTA (right). Numbers below images indicate the rank of the given gene in the list of correlated genes. See Figure S7 for expression patterns of the query genes. All images are scaled between 0 and 1 for visualization purposes. Key visible regions of high expression in Vim, Gfap, and Ctsd are circled in red for cross comparison of all images. (C) Gene Ontology term enrichment evaluated by Gorilla using the ranked correlated gene list produced by CoSTA (see Table S4). (D) Examples of gene expression images for genes highly ranked by CoSTA only (left), pixel only (middle), and both (right) as similar to Vim, Gfap, and Ctsd. Annotations next to gene names indicate rankings in CoSTA “C” and Pixel “P”. [file 12859_2021_4314_MOESM8_ESM.pdf]
