# Supplementary material for: CoSTA: unsupervised convolutional neural network learning for spatial transcriptomics analysis
Source: BMC Bioinformatics. 2021 Aug 9;22:397. doi: 10.1186/s12859-021-04314-1 (PMC8351440; doi:10.1186/s12859-021-04314-1)
Supplement: Supplementary file 13 — Additional file 13. Supplementary Table 1:. Comparison of CoSTA and SpatialDE classification of 10,000 simulated genes belonging to 5 spatial patterns (see Fig. 1). Normalized Mutual Information is used to measure the similarity between CoSTA or SpatialDE-derived cluster assignments and true cluster assignments (values closer to 1 indicate a higher concordance between true and predicted cluster memberships). At noise level 0.6, CoSTA performs better with the addition of center loss (0.91 vs. 0.52). For shuffled data, each gene matrix was identically shuffled as described in Fig. S12 and in the Methods. This shuffling preserves pixel correlations between genes but disrupts overall spatial patterns, allowing an evaluation of whether the tested analysis detects pixelwise or spatial information. [file 12859_2021_4314_MOESM13_ESM.pdf]

Supplementary Table 1

NMI	CoSTA		SpatialDE	
noise level (variance)	True data	Shuffled data	True data	Shuffled data
0.2	0.97	0.89	1	0.86
0.3	0.98	0.87	0.99	0.99
0.4	0.96	0.8	0.99	0.99
0.5	0.85	0.66	0.98	0.98
0.6	0.52|0.91	0.32	0.97	0.97
