# Supplementary material for: CoSTA: unsupervised convolutional neural network learning for spatial transcriptomics analysis
Source: BMC Bioinformatics. 2021 Aug 9;22:397. doi: 10.1186/s12859-021-04314-1 (PMC8351440; doi:10.1186/s12859-021-04314-1)
Supplement: Supplementary file 14 — Additional file 14. Supplementary Table 2: Clusters of SE genes identified by CoSTA in the MERFISH dataset (cell type patterns are included in clusters). [file 12859_2021_4314_MOESM14_ESM.pdf]

Supplementary Table 2

Gene	Cluster
Endothelial 1	0
Ermn	0
Gabra1	0
Gjc3	0
Igf1r	0
Man1a	0
Ndrg1	0
OD Mature 2	0
Sema3c	0
Sgk1	0
Slco1a4	0
Ttyh2	0
Aldh1l1	1
Amigo2	1
Ar	1
Arhgap36	1
Astrocyte	1
Cbln1	1
Cbln2	1
Cckar	1
Cpne5	1
Creb3l1	1
Crhr2	1
Cspg5	1
Dgkk	1
Excitatory	1
Gabrg1	1
Galr1	1
Glra3	1
Gpr165	1
Htr2c	1
Igf2r	1
Inhibitory	1
Irs4	1
Isl1	1
Kiss1r	1
Onecut2	1
Oprd1	1
Oprk1	1
Oprl1	1
Pak3	1
Pnoc	1
Prlr	1
Rnd3	1
Scg2	1
Slc17a6	1
Sox4	1
Sox6	1
Sox8	1
Syt4	1
Tmem108	1
Adora2a	2
Bdnf	2
Brs3	2
Ccnd2	2
Chat	2
Endothelia	2
Gbx2	2
Gem	2
Grpr	2
Krt90	2
Lpar1	2
Microglia	2
Nts	2
OD Mature	2
Rgs5	2
Rxfp1	2
Selplg	2
Cdkn1a	3
Cenpe	3
Cplx3	3
Cyp19a1	3
Fezf1	3
Fn1	3
Klf4	3
Mbp	3
Ndnf	3
Necab1	3
Ntng1	3
Nup62cl	3
OD Mature	3
Opalin	3
Plin3	3
Ramp3	3
Slc17a8	3
Sp9	3
Sytl4	3
Tacr1	3
Calcr	4
Cxcl14	4
Esr1	4
Gad1	4
Gal	4
Gda	4
Npy2r	4
Penk	4
Rgs2	4
Serpinb1b	4
Th	4
Trhr	4
Coch	5
OD Immat	5
Pcdh11x	5
Pdgfra	5
Traf4	5
Crhbp	6
Cyr61	6
Ebf3	6
Endothelia	6
Fst	6
Gnrh1	6
Lmod1	6
Mki67	6
Myh11	6
OD Immat	6
OD Mature	6
Oxt	6
Pericytes	6
Sst	6
Syt2	6
Tac2	6
Ucn3	6
Adcyap1	7
Aqp4	7
Avpr1a	7
Cckbr	7
Cd24a	7
Ependyma	7
Etv1	7
Fos	7
Mlc1	7
Nnat	7
Nos1	7
Npy1r	7
Omp	7
Pou3f2	7
Sema4d	7
Slc15a3	7
Slc18a2	7
Sln	7
Tac1	7
Tiparp	7
Avpr2	8
Egr2	8
Galr2	8
Pgr	8
Synpr	8
Vgf	8
