# Supplementary material for: CoSTA: unsupervised convolutional neural network learning for spatial transcriptomics analysis
Source: BMC Bioinformatics. 2021 Aug 9;22:397. doi: 10.1186/s12859-021-04314-1 (PMC8351440; doi:10.1186/s12859-021-04314-1)
Supplement: Supplementary file 15 — Additional file 15. Supplementary Table 3: CoSTA was applied to gene images from 4 different Slide-seq experiments and evaluated for whether it could separate gene images correctly into which original tissue slice (overall pattern) they came from. The table shows the confusion matrix of clustering labels derived from CoSTA results compared to the original known experimental label. [file 12859_2021_4314_MOESM15_ESM.pdf]

Supplementary Table 3

	**0**	**1**	**2**	**3**
**0**	2266	36	2	6
**1**	1	5117	115	157
**2**	0	78	7396	102
**3**	4	114	91	7085

Clustering label

Experiment label
