# Supplementary material for: CoSTA: unsupervised convolutional neural network learning for spatial transcriptomics analysis
Source: BMC Bioinformatics. 2021 Aug 9;22:397. doi: 10.1186/s12859-021-04314-1 (PMC8351440; doi:10.1186/s12859-021-04314-1)
Supplement: Supplementary file 18 — Additional file 18. Supplementary Table 6: Runtime of CoSTA for 3-day and 2-week Slide-seq data. Runtimes are measured in minutes and under different numbers of clusters being assigned during training. [file 12859_2021_4314_MOESM18_ESM.pdf]

Supplementary Table 6

(running with 30 clusters)

**Slide-seq**	**3-day**	**2-week**
# of genes	7576	7294
size of image	48X48	48X48
runtime (in min)	11.5	12.5

(running with assigning different clusters)

2-week	10 clusters	20 clusters	30 clusters	50 clusters	75 clusters	100 clusters
runtime (in min)	8	9.5	12.5	14.5	21	29.5

CPU	Intel i9-9880H
Memory	64GB
GPU	NVIDIA Quadro T2000
